# Genome-wide analysis of the WRKY gene family unveil evolutionary history and expression characteristics in tomato and its wild relatives

**DOI:** 10.3389/fgene.2022.962975

**Published:** 2022-09-15

**Authors:** Guan Liu, Dongye Zhang, Tingting Zhao, Huanhuan Yang, Jingbin Jiang, Jingfu Li, He Zhang, Xiangyang Xu

**Affiliations:** ^1^ College of Advanced Agriculture and Ecological Environment, Heilongjiang University, Harbin, China; ^2^ State Key Laboratory of Tree Genetics and Breeding, College of Forestry, Northeast Forestry University, Harbin, China; ^3^ Laboratory of Genetic Breeding in Tomato, College of Horticulture and Landscape Architecture, Northeast Agricultural University, Harbin, China

**Keywords:** genome-wide, WRKY transcription factor, tomato, whole genome duplication, tandem duplication, expression divergence

## Abstract

WRKY transcription factors (WRKYs) are one of the largest plant gene families in plants involved in various biotic and abiotic stress responses. Based on the conservation of WRKY proteins, we identified a total of 642 WRKYs in *Amborella trichopoda* (33), *Vitis vinifera* (64), *Arabidopsis thaliana* (48), *Solanum lycopersicoides* (88), *S. pennellii* (77), *S. pimpinellifolium* (80), *S. lycopersicum* var. cerasiforme (85), *S. lycopersicum* cv. Heinz1706 (85), and *S. lycopersicum* cv. M82 (82) genomes. Phylogenetic analysis clustered WRKYs from nine genomes above into two clusters (Cluster1 and Cluster2). Evolutionary analysis revealed that most of the WRKYs in tomato and its wild relatives were expanded after the whole genome triplication (WGT) event of *Solanum* ancestor. Effects of tandem duplication (TD) event for WRKYs revealed that several WRKYs have experienced TD event and drove the expansion of the WRKY gene family in tomato and its wild relatives. Comparative analysis of WRKYs derived from WGT and TD events indicated that the WGT event performed a stronger influence on the expansion of the WRKY gene family than the effects of the TD event. Transcriptome profiling of WRKYs in *S. lycopersicum* cv. Heinz1706 under the biotic stress condition relative to the control condition uncovered a number of up-regulated WRKYs in response to biotic stress. The diversified expression pattern among paralogs derived from TD and WGT implied the impact of gene duplication events on gene functional divergence and diversity in tomato. We hope that this project will supply novel knowledge for studying the evolutionary history and functional characteristics of WRKYs involved in biotic stress in tomato.

## Background

The WRKY gene family is one of the largest transcription factor families modulating plant development and growth, in particular the responses to biotic and abiotic stresses. The primary characteristic of WRKY proteins is the DNA-binding domain, which contains the WRKYGQK sequence and a zinc-binding motif (Bakshi and Oelmüller, 2014). So far, WRKYs were found in a wide range of higher plants families using the domain conservation, including the 74 WRKYs identified in *Arabidopsis*, the total of 197, 119, and 100 WRKYs in soybean, maize, and rice, as well as a number of WRKYs in horticultural crops including strawberry (62), apple (127), citrus (50), and etc (Rushton et al., 2010; Wei et al., 2012; Ayadi et al., 2016; He et al., 2016; Meng et al., 2016; Wei et al., 2016). These WRKYs fell into three distinct groups (group I, group II, and group III) (Li et al., 2011). In addition to the genome-wide structural characterization of WRKYs in these different species, WRKYs have also been functionally characterized regarding their important roles involving the defense response to several pathogens and abiotic stresses (heat, drought, salinity, and oxidative stresses) (Joshi et al., 2016). For instance, WRKY27 in pepper, WRKY39, and WRKY40 in upland cotton modulate the resistance to *R. solanacearum* and wounding-induced response (Shi et al., 2014; Wang et al., 2014; Hussain et al., 2019). In *Arabidopsis*, WRKY25, WRKY33, and WRKY34 were up-regulated in response to cold and salt treatment respectively along with enhanced stress tolerance in the overexpression line, indicating their roles in conferring tolerance to abiotic stresses (Li et al., 2011). Thus, genomic analysis and functional studies regarding the roles of WRKYs in responses to abiotic and biotic stresses are critical to improving plant resilience and enhancing plant production in face of the changing environments.

Tomato (*Solanum lycopersicum*) is a worldwide economically important vegetable crop due to its high-level nutrients of fleshy fruit. In 2012, the first tomato high-quality genome, inbred cultivar “Heinz 1706,” was sequenced and released (Sato et al., 2012). After that, genomes of three tomato wild relatives, *S.lycopersicoides*, *S. pennellii*, and *S. pimpinellifolium*, as well as two different phenotypes, *S. lycopersicum* var. cerasiforme and *S. lycopersicum* cv. M82 were sequenced, which provided an extensive genomic resource for comparative genomic studies in the *Solanum* lineage (Bolger et al., 2014; Wang et al., 2020; Takei et al., 2021; Powell et al., 2022). Comparison between *S. lycopersicum* cv. Heinz1706 and *V. vinifera* genomes revealed that *S. lycopersicum* cv. Heinz1706 and *V. vinifera* shared the whole genome triplication (WGT) with common eudicot ancestor, followed by a recent WGT in *S. lycopersicum* cv. Heinz1706 (Sato et al., 2012). These comparative frameworks provided an invaluable opportunity for scientists to study the evolutionary relationship of the impact of gene duplications to gene function diversification and evolution.

Here, we collected three tomato species including *S. lycopersicum* var. cerasiforme, *S. lycopersicum* cv. Heinz1706, and *S. lycopersicum* cv. M82, and three tomato wild relatives including *S. lycopersicoides*, *S. pennellii*, *S. pimpinellifolium* to perform genome-wide comparative genomics analysis of WRKY gene family among six *Solanum* genomes. Then, we collected one basal angiosperm, *A. trichopoda*, one diploid species after WGT of Eudicot ancestor, *V. vinifera*, and one model plant, *A. thaliana* as control, to dissect the evolutionary history and gene family expansion of WRKYs in *Solanum* lineage. From comparison between *V. vinifera* and six *Solanum* genomes respectively, we analyzed the influence of a WGT event on the WRKY gene family to investigate the ancient loci or gene orders retained in six *Solanum* genomes. Further, we performed the analysis of tandem duplication (TD) events to detect the influence on the expansion of the WRKY gene family in six *Solanum* genomes. Finally, we investigated the expression patterns of WRKY TFs in *S. lycopersicum* cv. Heinz1706, in particular the WRKYs involved in the recent WGT and TD events.

## Materials and methods

### Data resource

*V. vinifera* (PN40024.v4) and *A. trichopoda* (AMTR1.0) genomic data were downloaded from Ensembl Genomes 53 (https://plants.ensembl.org/) (Howe et al., 2020). *A. thaliana* (TAIR11) genomic data were downloaded from TAIR (https://www.arabidopsis.org/) (Cheng et al., 2017). *S. lycopersicoides*, *S. pennellii*, *S. pimpinellifolium*, *S. lycopersicum* var. cerasiforme, *S. lycopersicum* cv. Heinz1706, and *S. lycopersicum* cv. M82 genomic data were downloaded from Sol Genomics Network (https://solgenomics.net/) (Fernandez-Pozo et al., 2015). The profile Hidden Markov Models (HMMs) of the WRKY domain (PF03106.18) was downloaded from Pfam 33.1 (May 2020, 18,259 entries) (http://pfam.xfam.org/) (Mistry et al., 2021). The RNA-seq data of *S. lycopersicum* cv. Heinz1706 was downloaded from SRA-NCBI with accession number: PRJNA378182 (Huot et al., 2018).

### Identification of WRKYs

The “hmmsearch” module with “trusted cutoff” as threshold in HMMER v3.2.1 (http://hmmer.org/) was employed to identify the putative WRKY transcription factors in *V. vinifera*, *A. trichopoda*, *A. thaliana*, and six *Solanum* genomes. The highly conserved WRKY proteins with E-value =<1e−05 in the results from HMMER software were selected to construct the species-specific profile HMMs for nine target genomes with “hmmbuild” module in HMMER v3.2.1. The new species-specific profile HMMs of nine target genomes were used to search their corresponding genome protein sequences to get the WRKY transcription factor candidates among nine target genomes. The InterProScan was used to validate the WRKY domain among nine target genomes (Jones et al., 2014).

### Reconstruction of phylogenetics among different species

All protein sequences of WRKY transcription factors among nine genomes retrieved from respective genome annotations were performed with multiple sequence alignments (MSA) with the “Clustal-W” module in MEGA version 10.2.5 (Kumar et al., 2018). The MSA file was used to construct a phylogenetic tree with the maximum likelihood (ML) statistical method and Jones-Taylor-Thornton (JTT) substitution model. The phylogenetic tree of WRKY transcription factors among *V. vinifera* and six *Solanum* genomes was performed with identical procedures and parameters.

### Analysis of tandem duplicated WRKYs

For each genome among the nine target genomes, the bidirectional BLAST of protein sequences with diamond blastp was employed to identify paralogous gene pairs with E-value cutoff =< 1e−20 (Buchfink et al., 2021). The paralogous gene pairs with high similarity were detected in their location on pseudo-molecular chromosomes. The closer paralogous protein-coding genes within the identical genomic region was a tandem array, and the members of the tandem array were tandem duplicated genes in one corresponding genome. Any two tandem duplicated WRKYs within one tandem array were calculated as the ratio of the rates of non-synonymous to synonymous substitutions (Ka/Ks) using KaKs_Calculator 2.0 (Wang et al., 2010).

### Analysis of whole-genome duplication in tomato relative to *V. vinifera*

The MCscanX toolkit was used to detect the orthologous genomic regions between *V. vinifera* and six *Solanum* genomes respectively (Wang et al., 2012). Firstly, the BLAST of protein sequences with diamond blastp was employed to identify orthologous gene pairs with E-value cutoff =< 1e−20 between the different genomes (Buchfink et al., 2021). Secondly, the MCscanX toolkit was employed to identify orthologous regions with the parameters (MATCH_SIZE = 5 and E_VALUE = 1e-20) between *V. vinifera* and six *Solanum* genomes, respectively. Based on the identified WRKY transcription factors in *V. vinifera* and six *Solanum* genomes, the orthologous genomic regions between *V. vinifera* and six *Solanum* genomes were parsed to get the WRKY orthologous gene pairs, which might be generated by a WGT event.

### Analysis of transcriptomic expression

Transcriptomic data in this study was derived from the NCBI sequence read archive (SRA, bio-project No: PRJNA378182) (Kodama et al., 2012). A total of nine samples with three replicates for each from an experiment set up with three conditions (control, Lso-positive psyllids, Lso-negative psyllids) over three timepoints (Week1, Week2, and Week4) after treatment was adopted to study the roles of WRKYs in response to biotic stresses (CITE) (Huot et al., 2018). Based on released information, all samples were sequenced by the Illumina HiSeq 2500 platform with an average of 24.5 million reads per sample. For each sample, we quantified the count of reads based on gene annotations by salmon pipeline (lib-type: single-end). Reads count from each sample were further normalized using DESeq2 with default settings to eliminate effects of different library sizes before quantification of gene expression (Love et al., 2014). We quantified the relative transcripts abundance by transcripts per million (TPM) and performed the Pearson-correlation analysis over replicates for each sample to confirm the reproducibility of our experiments (Patro et al., 2017). Replicates with low correlation (*r* < 0.8, *p* < 0.05) was discarded for analysis. Average TPMs over replicates with high correlation were calculated as respective expression values. To compare the relative expression of WRKYs over time points and treatments, TPMs were further transformed into Z-score for heatmap visualization by the Pheatmap function in R (RRID:SCR_016418).

## Results

### The larger WRKY gene family in *Solanum* genomes

**FIGURE 1:**
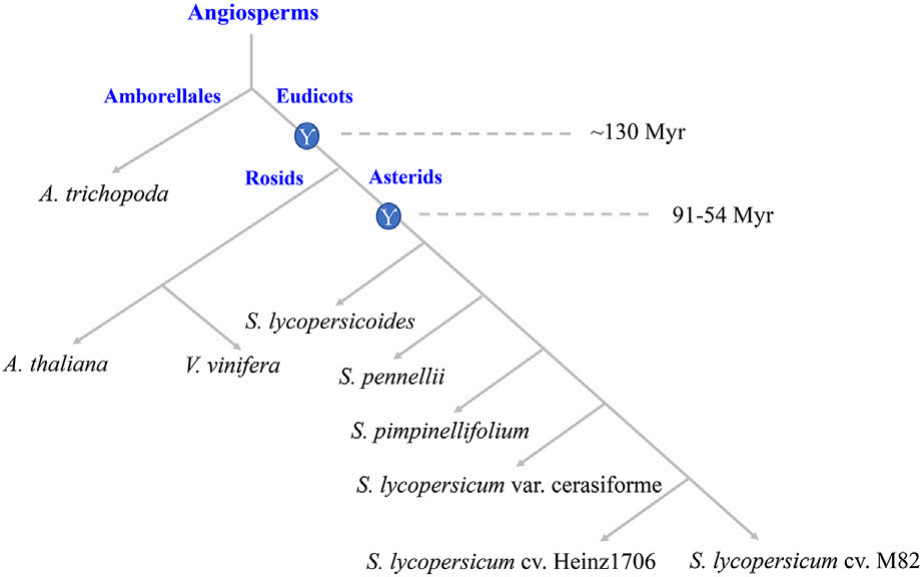
Evolutionary relationship and ancestral polyploid events. The circles with Ƴ represent the WGT event of Eudicot ancestor and *Solanum* lineage.

High-throughput genome sequencing technology in plants facilitates the analyses of genome evolution and genome-wide identifications of gene families. *A. trichopoda* was one species of the basal angiosperm without the recent and lineage-specific genome duplications, which provided the basis for studying the evolution of plant polyploidy (DePamphilis et al., 2013). *V. vinifera* was one species of the rosids have not undergone recent genome duplication after the Eudicot WGT (Jaillon et al., 2007). *A. thaliana* was the diploid model plant, and its genomic data was interpreted by researchers comprehensively (The Arabidopsis Genome Initiative, 2000). The evolutionary relationship among genomes of *S. lycopersicum* var. cerasiforme, *S. lycopersicum* cv. Heinz1706, and *S. lycopersicum* cv. M82 and their wild relatives, *S. lycopersicoides*, *S. pennellii*, *S. pimpinellifolium* are shown in (Figure 1). Based on the conservation of the DNA binding domain from WRKYs, we identified 33, 64, 48, 88, 77, 80, 85, 85, and 82 WRKYs in *A. trichopoda*, *V. vinifera*, *A. thaliana*, *S. lycopersicoides*, *S. pennellii*, *S. pimpinellifolium*, *S. lycopersicum* var. cerasiforme, *S. lycopersicum* cv. Heinz1706, and *S. lycopersicum* cv. M82, respectively. So, from basal angiosperm to *Solanum* lineage, the copy numbers of WRKYs increased.

To better understand the distribution of WRKYs in genome, we characterized the genomic locations of these WRKYs. Among six *Solanum* genomes, the Chr05 pseudo-molecular chromosome in *S. pennellii*, *S. pimpinellifolium*, *S. lycopersicum* cv. Heinz1706, and *S. lycopersicum* cv. M82 contained the most WRKY transcription factors compared to the rest pseudo-molecular chromosomes (Table 1). The chr08 contained the most WRKY transcription factors in *S. lycopersicoides* genome, with chr10 in *S. lycopersicum* var. cerasiforme genome. Notably, None WRKY was identified in Chr11 pseudo-molecular chromosome for the six *Solanum* genomes. This is consistent with the previous report. Except that, the previous report identified 83 WRKYs in tomato with complex sequence retrieval from public databases, which had a slight difference in numbers of WRKYs with our analysis. This might be attributed to the update of tomato genome annotation.

**TABLE 1:** Summary of WRKYs in tomato and its relatives.

Categories	*S. lycopersicoides*	*S. pennellii*	*S. pimpinellifolium*	*S. lycopersicum*		
				var. *cerasiforme*	cv. *Heinz1706*	cv. *M82*
Chr01	7	7	7	7	7	7
Chr02	11	9	8	9	9	9
Chr03	8	9	8	8	8	8
Chr04	5	5	8	8	7	7
Chr05	10	12	15	10	19	17
Chr06	6	5	4	5	5	5
Chr07	8	7	5	6	7	7
Chr08	14	8	8	9	7	6
Chr09	5	4	4	4	4	4
Chr10	6	5	6	13	5	5
Chr11	0	0	0	0	0	0
Chr12	6	6	7	6	7	7
Unknown	2	0	0	0	0	0
Total	88	77	80	85	85	82

### Phylogenetic analysis of WRKYs in different genomes

**FIGURE 2:**
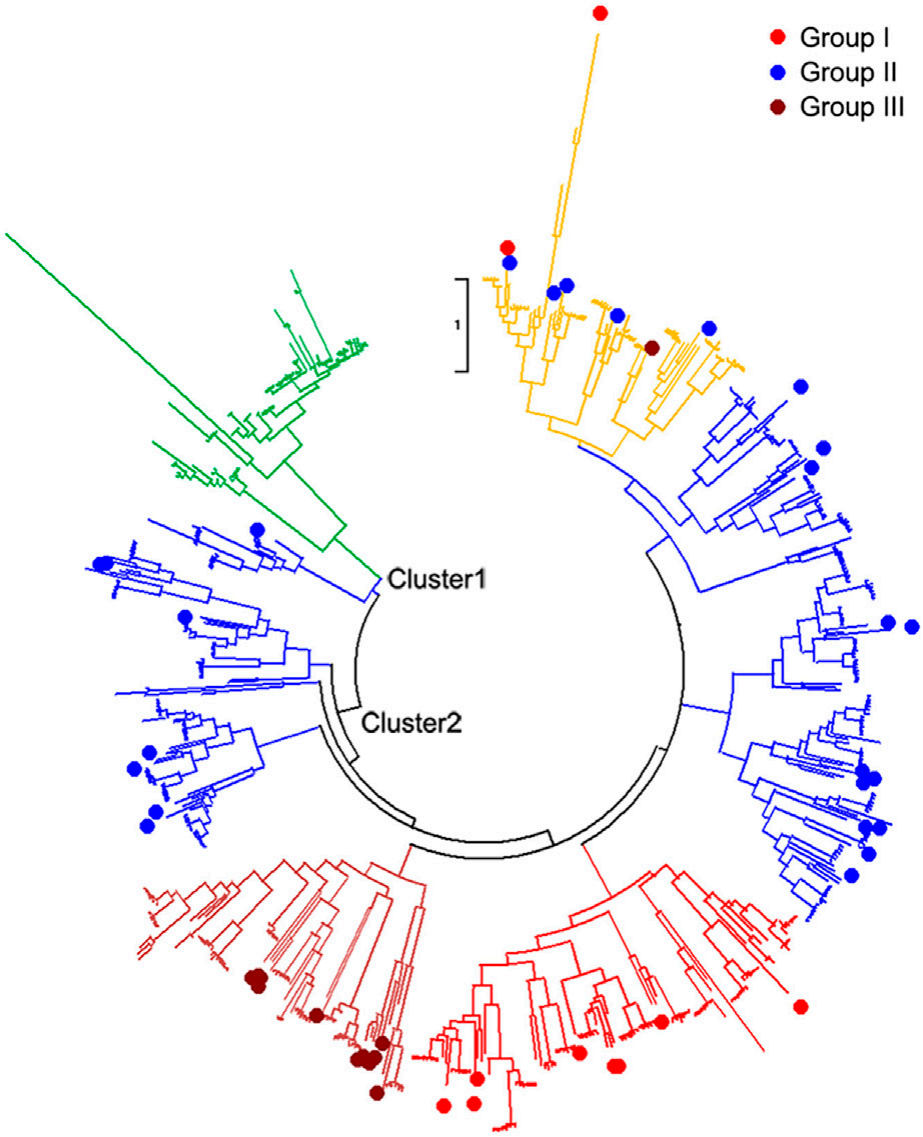
Phylogenetic tree of WRKYs in *A. trichopoda*, *V. vinifera*, *A. thaliana*, and six *Solanum* genomes. Red, blue, and blown colors of tree branches, as well as the corresponding solid circles, represent the group I, group II, and group III of *A. thaliana* WRKYs group. The green color of tree branches represents the *Solanum* specific WRKY group. The yellow color of tree branches represents the mixed group of the group I, group II, and group III of *A. thaliana* WRKYs group. The Cluster1 and Cluster2 within phylogenetic tree represent different clusters.

All 642 protein sequences of the identified WRKYs among nine genomes were used to reconstruct the phylogenetic tree. From the phylogeny, all the identified WRKYs were grouped into two different clusters including Cluster1 and Cluster2 (Figure 2). Cluster1 contained *Solanum*-specific WRKYs group and part of group II of *Arabidopsis* WRKYs. In *Solanum* specific WRKY group, there are 4, 9, 15, 16, 15, and 13 WRKYs distributed into *S. lycopersicoides*, *S. pennellii*, *S. pimpinellifolium*, *S. lycopersicum* var. cerasiforme, *S. lycopersicum* cv. Heinz1706, and *S. lycopersicum* cv. M82 genomes, respectively, indicating that these WRKYs might be the products accompanied after the evolution of the *Solanum* ancestor (Table 2). In total, 72 *Solanum* specific WRKYs were obtained, which represented 14.5% of total WRKYs (497) in six *Solanum* genomes. Out of *Arabidopsis* WRKY Group I, II, and III, Group III contained the most WRKYs (286), representing 44.5% of total WRKYs in *A. trichopoda*, *V. vinifera*, *A. thaliana*, and six *Solanum* genomes. Interestingly, there is a group mixed with the members of *Arabidopsis* WRKY Group I, II, and III. This mixed group contained 78 WRKYs distributed into the analyzed nine genomes, representing 11.2% of total WRKYs in *A. trichopoda*, *V. vinifera*, *A. thaliana*, and six *Solanum* genomes.

**TABLE 2:** Statistics of WRKYs are distributed into different *A. thaliana* WRKY groups.

Categories	Group I	Group II	Group III	Mixed group	*Solanum*-specific group
*A. trichopoda*	6	18	4	5	0
*A. thaliana*	8	20	12	8	0
*V. vinifera*	15	33	6	10	0
*S. lycopersicoides*	18	43	14	9	4
*S. pennellii*	15	34	10	9	9
*S. pimpinellifolium*	13	33	9	10	15
*S. lycopersicum* var. cerasiforme	13	35	12	9	16
*S. lycopersicum* cv. Heinz1706	15	35	11	9	15
*S. lycopersicum* cv. M82	15	35	10	9	13
Total	118	286	88	78	72

### Evolution of WRKYs in tomato and its wild relatives

**FIGURE 3:**
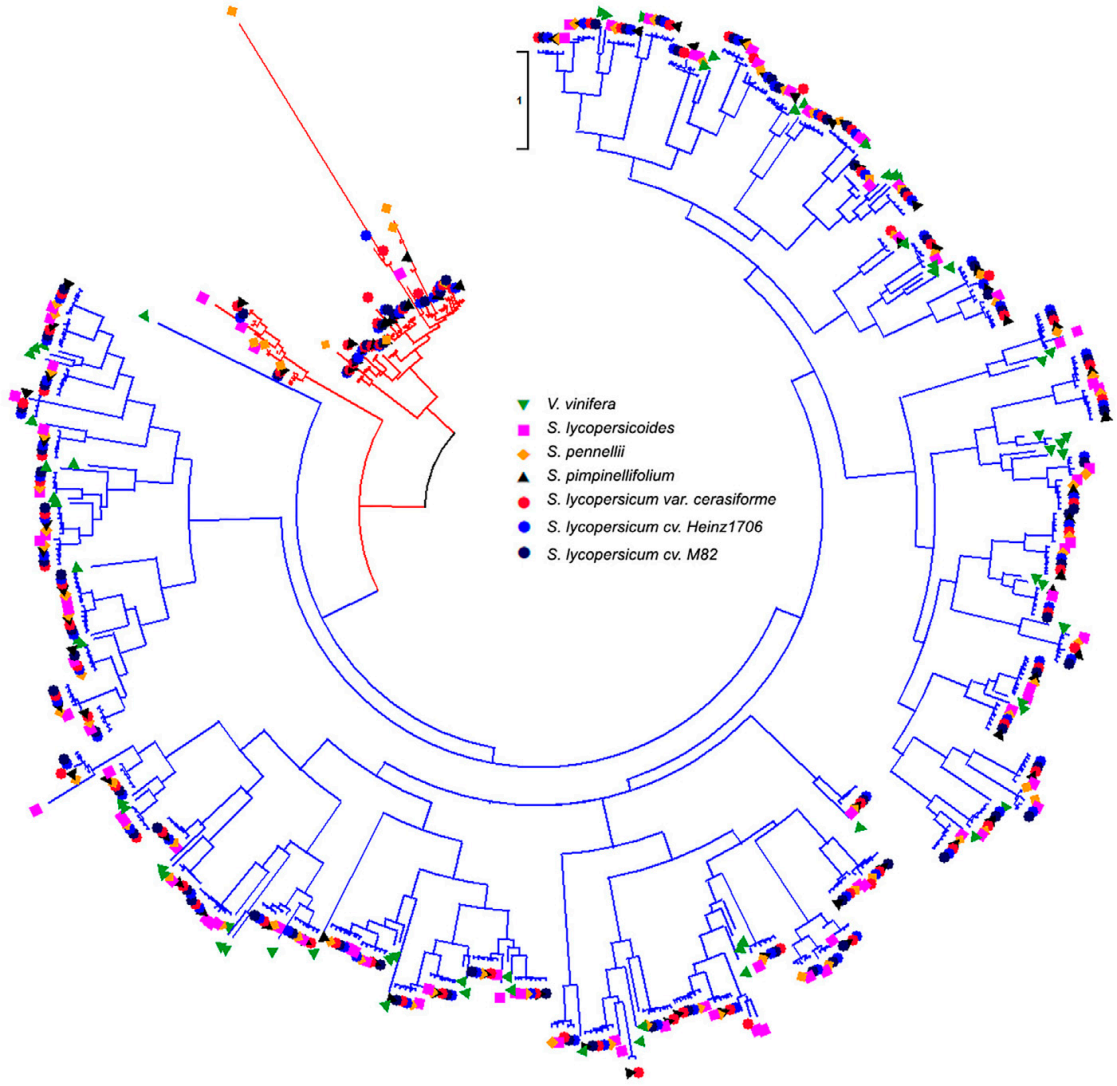
Phylogenetic tree of WRKYs in *V. vinifera* and six *Solanum* genomes. Red and blue colors of tree branches represent the *Solanum* specific WRKYs group and the *Solanum* shared group with *V. vinifera*. Green lower solid triangle, orange solid square, yellow solid diamond, black upper solid triangle, red solid circle, blue solid circle, and black solid circle represent the WRKYs in *V. vinifera*, *Solanum lycopersicoides*, *S. pennellii*, *S. pimpinellifolium*, *S. lycopersicum* var. cerasiforme, *S. lycopersicum* cv. Heinz1706, and *S. lycopersicum* cv. M82 genomes.

A previous report revealed that the *Solanum* lineage experienced the recent WGT event after the WGT event of Eudicots ancestor shared with rosids, but *V. vinifera* genome did not experience a WGT event after that (Jaillon et al., 2007). This result demonstrated that six *Solanum* genomes had three copies of orthologous genomic regions compared with *V. vinifera* genome, which could detect the ancient gene loci or gene orders in the genomes of tomato and its wild relatives. Thus, we performed the detection of WRKYs derived from the recent WGTs to study the copy-number variations of WRKY in *Solanum* genomes compared with *V. vinifera*. We collected 561 identified WRKYs among *V. vinifera* and six *Solanum* genomes to detect their evolutionary relationship (Figure 3). The *V. vinifera* WRKYs exhibited irregular distributions within different WRKY groups, and even grouped together. We compared *V. vinifera* genome to six *Solanum* genomes respectively to investigate the effects of WGT for the WRKYs in tomato and its wild relatives. After the implementation of syntenic analysis between the *V. vinifera* and six *Solanum* genomes, we obtained 50 orthologous gene pairs between *V. vinifera* and *S. lycopersicoides* genomes covering 40 and 50 WRKYs in *V. vinifera* and *S. lycopersicoides*, respectively. This result indicated that three copy paralogous WRKYs in *S. lycopersicoides* genome experienced genes loss after WGT event, along with two three-copies retained, six two-copies retained, and 32 one-copies retained in *S. lycopersicoides* genome (Table 3). For the comparison between *V. vinifera* and *S. pennellii* genomes, we obtained 46 orthologous gene pairs containing 39 and 46 WRKYs in *V. vinifera* and *S. pennellii* genomes, which included seven two-copies retained and 33 one-copies retained in *S. pennellii* genomes. For the comparison between *V. vinifera* and *S. pimpinellifolium* genomes, we obtained 39 orthologous gene pairs containing 33 and 39 WRKYs in *V. vinifera* and *S. pennellii* genomes, which included one three-copies retained, four two-copies retained, and 28 one-copies retained in *S. pennellii* genomes. For the three tomatoes, 38, 39, and 38 *V. vinifera* WRKYs were detected 45, 48, and 45 orthologous WRKYs in *S. lycopersicum* var. cerasiforme, *S. lycopersicum* cv. Heinz1706, and *S. lycopersicum* cv. M82 genomes, respectively.

**TABLE 3:** Statistics of whole-genome duplicated WRKYs among six *Solanum* genomes.

Categories	Total WRKYs	No. of whole-genome duplicated WRKYs	Percentage (%)	No. of three-copies retained	No. of two-copies retained	No. of one-copy retained
*S. lycopersicoides*	88	50	56.82	2	6	32
*S. pennellii*	77	46	59.74	0	7	32
*S. pimpinellifolium*	80	39	48.75	1	4	28
*S. lycopersicum* var. *cerasiforme*	85	45	52.94	1	5	32
*S. lycopersicum* cv. *Heinz1706*	85	48	56.47	1	7	31
*S. lycopersicum* cv. *M82*	82	45	54.88	1	5	32

### Effects of TD event for WRKYs in tomato and its relatives

TD event will increase the gene copy number and further lead to the expansion of gene family in plants (Jaillon et al., 2007). To address the expansion of WRKY gene family in tomato and its relatives, we analyzed the influence of TD events on the WRKY gene family in six *Solanum* species. Combining sequence similarity and gene location, we got 4, 3, 4, 3, 5, and 4 WRKY tandem arrays containing 9, 6, 8, 6, 10, and 8 WRKYs which represented 10.2%, 7.8%, 10%, 7%, 11.8%, and 9.8% of corresponding total WRKYs in *S. lycopersicoides*, *S. pennellii*, *S. pimpinellifolium*, *S. lycopersicum* var. cerasiforme, *S. lycopersicum* cv. Heinz1706, and *S. lycopersicum* cv. M82 genomes, respectively (Table 4). Out of six *Solanum* genomes, *S. pimpinellifolium*, *S. lycopersicum* cv. Heinz1706, and *S. lycopersicum* cv. M82 genomes only contained two-copies of tandem arrays.

**TABLE 4:** Statistics of tandem duplicated WRKYs and tandem arrays among six *Solanum* genomes.

Categories	Total WRKYs	No. of tandem duplicated WRKYs	No. of tandem arrays	Percentage (%)
*S. lycopersicoides*	88	9	4	10.23
*S. pennellii*	77	6	3	7.79
*S. pimpinellifolium*	80	8	4	10
*S. lycopersicum* var. *cerasiforme*	85	6	3	7.06
*S. lycopersicum* cv. *Heinz1706*	85	10	5	11.76
*S. lycopersicum* cv. *M82*	82	8	4	9.76

To further investigate the evolution of WRKY tandem arrays, we combined the analyses between the WRKYs from WGT and TD events. In *S. lycopersicoides* genome*, V. vinifera* WRKY (Vitvi07g00026) corresponded to a single orthologous gene (Solyd09g065590), which meant that this WRKY only had one copy retained after WGT event (Supplementary Table S1). But this gene (Solyd09g065590) in *S. lycopersicoides* genome experienced TD event and retained three-copies in a tandem array (Solyd09g065590, Solyd09g065600, and Solyd09g065610) (Supplementary Table S2). Out of the three members of this tandem array, Solyd09g065590 and Solyd09g065610 were all WRKYs, but Solyd09g065600 was a short protein with126 amino-acids and an incomplete WRKY domain. In *S. lycopersicum* var. cerasiforme genome, *V. vinifera* WRKY (Vitvi02g00114) had three orthologous genes (SLYcer08g00549, SLYcer08g06424, and SLYcer10g00730), which meant that the orthologous genes of *V. vinifera* WRKY (Vitvi02g00114) had three copies retained after WGT event. In *S. lycopersicum* cv. M82 genome, three orthologous genes (Solyc01g095630.3, Solyc05g050330.3, and Solyc10g009550.3) retained after WGT event relative to their ortholog *V. vinifera* WRKY (Vitvi15g01003). Also, the gene (Solyc05g050330.3) experienced TD event and generated a two-copies tandem array (Solyc05g050330.3 and Solyc05g050340.4).

Through the comparison of tandem duplicated WRKY gene pairs between *V. vinifera* and six *Solanum* genomes, we investigated that the tandem duplicated WRKYs in six *Solanum* genomes did not have the corresponding tandem duplicated genes in *V. vinifera* genome, which meant that the tandem duplicated WRKYs in six *Solanum* genomes were generated after the divergence between *V. vinifera* and *Solanum* ancestor. To investigate the selection pressures among 47 tandem duplicated WRKYs, we obtained 34 tandem duplicated WRKY gene pairs, and 12 WRKY gene pairs indicated to experience positive selection, with 21 WRKY gene pairs undergoing negative selection (Supplementary Table S3).

### Global transcriptome profiling of WRKYs in response to biotic stress

**FIGURE 4:**
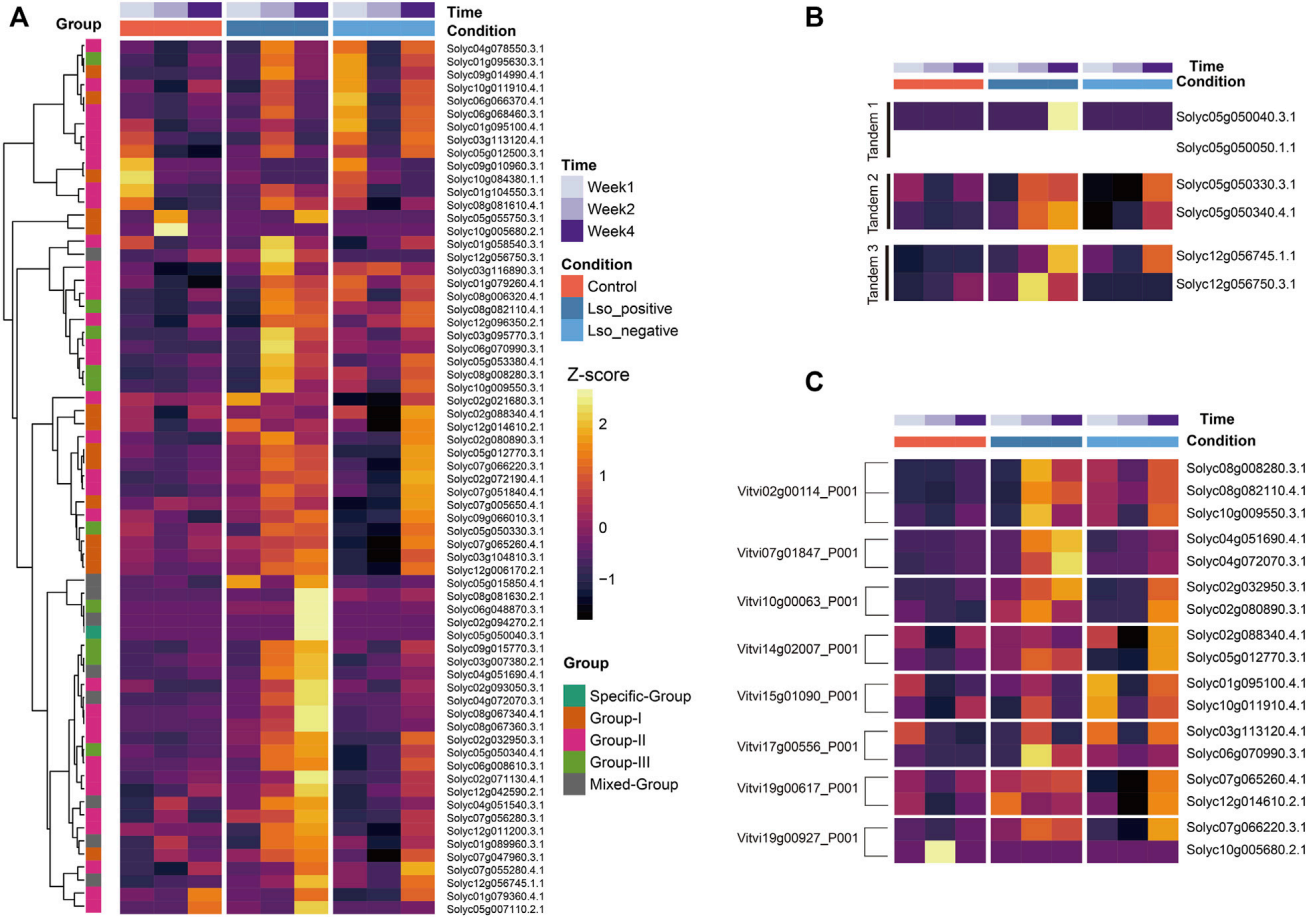
Expression profile of WRKYs identified in *S. lycopersicum* cv. Heinz1706 genome. **(A)** Hierarchical-Clustering of 62 WRKYs using Z-score transformed TPM data. The meta feature including time, condition, group of WRKYs were marked in legend for reference. **(B)** Five WRKYs derived from TD event were displayed to compare expression among samples under control and treatment condition. **(C)** Expression of eight groups of WRKYs paralogs derived from the recent WGT event were displayed in heatmap.

To identify biotic stress-responsive WRKY members in *S. lycopersicum* cv. Heinz1706, we referred to the publicly available RNAseq datasets including experiments under both biotic stress treatment and control conditions. After data processing, an average of 67.84% of reads were mapped to tomato transcripts using the salmon pipeline (Patro et al., 2017). Filtering of transcripts (counts >0) left 26,391 out of 33,950 transcripts for normalizations which include 63 out of 82 WRKY transcripts from *S. lycopersicum* cv. Heinz1706 reference genome. Both (variance stabilizing transformation (VSD) and transcripts per million (TPM) quantification based on normalized read counts per library were conducted to estimate the replicability of the experiment, and all samples are in high confidence correlation (*r* < 0.8) (Supplementary Figure S1). A hierarchical-clustering heatmap based on Z-score transformed TPMs clearly clustered WRKYs with eminent variations of stress responses over timepoints and two treatment types. Particularly, a great portion of WRKYs from Group II up-regulated at the 2 weeks after treatment of Lso-positive psyllids. A small cluster of WRKYs which contains six genes (as shown from the first six rows of heatmap) were identified with pronounced elevated expressions after the first week of Lso-negative psyllids treatment and these small subsets of genes might involve the early response to biotic stresses. And five WRKYs were uniquely up-regulated at the 4 weeks after Lso-positive psyllids treatment, including three WRKYs from the mixed group (Solyc05g015850.4, Solyc08g081630.2, and Solyc02g094270.2), one specific group (Solyc05g050040.3), and one form Group III (Solyc06g048870.3) (Figure 4A). Lastly, a great number of WRKYs exhibited high-level responses both the 2 and 4 weeks after experiments which might be explained by their functional redundancy.

### Transcriptional divergence of WRKY paralogs derived from gene duplications

To uncover the evolutionary impacts of gene duplications on gene expression of WRKYs in tomato, we examined the expression profiles of tandem duplicated gene pairs (TD) and genes derived from a WGT event. As especially shown in our selected transcriptome dataset, there were five groups of tandem duplicated genes (10 WRKYs) along with expressions from three groups (Figure 4B). The clustering heatmap of expression clearly exhibited the expression variations between each two tandem duplicated genes. As a contrast with the tandem 1 group, Solyc05g050050.1.1 were completely not expressed among all timepoints and conditions while its tandem duplicated paralog Solyc05g050040.3 highly expressed in response to Lso-positive psyllids treatment at the week 2. We also noticed the variations from tandem group 3 where Solyc12g056750.3 down-regulated at week 2 under the Lso-positive psyllids compared to week 4 under both the Lso-negative/positive psyllids. However, its paralogs Solyc12g056745.1 exhibited an adverse regulation pattern. These expression profile variations implied the functional variations during the expansion of WRKY TFs derived from TD event.

Similarly, we compared the transcription profiles of 17 WRKYs derived from the WGT event (Figure 4C). Among three paralogous WRKYs identified in *S. lycopersicum* cv. Heinz1706 relative to their *V. vinifera* ortholog Vitvi02g00114, two WRKYs (Solyc08g008280.3 and Solyc10g009550.3) presented the high-consensus level expression profile while the uniquely elevated expression at the week 4 positive treatment was identified from the rest paralog Solyc08g082110.4. For the rest seven orthologous groups relative to *V. vinifera* WRKYs, expression variations at the diverse levels in between respective paralogous genes were identified, indicating that both functional redundancy and sub-functionalization occurred during the WRKYs evolution, and both WGT and TD events might be the forces leading to functional divergence among WRKYs in *S. lycopersicum* cv. Heinz1706.

## Discussion

### Classification of WRKYs in *Solanum* lineage

The primary characteristics of WRKY proteins are the DNA-binding domain, which contains the WRKYGQK sequence and a CX4–5CX22–23HXH zinc binding (Bakshi and Oelmüller, 2014). Based on the numbers of the WRKY domain, WRKY proteins could be divided into different groups. WRKY proteins from group I are encoded by two WRKY domains and only one domain was found from the remaining two WRKY groups. These two groups were further classified based on the presence of C2-H2 (C-X4-5-CX22-23-H-X1-H) motif from group II and the C2-HC (C-X7-C-X23-H-X-C) zinc finger motif from group III (Li et al., 2011). The WRKY gene family was illustrated comprehensively in *A. thaliana* (Rushton et al., 2010). In this project, we combined the phylogeny analysis with full-length protein sequences and the classification of WRKY groups in *A. thaliana* to classify the WRKY groups in *Solanum* lineage. Generally, highly conserved WRKYs will group together, and perform similar or identical molecular functions, belonging to the same WRKY group. So, the *Solanum* WRKYs were clustered together with *A. thaliana* WRKY groups I, II, and III, named *Solanum* WRKYs groups I, II, and III. In Cluster1, there is a group without *A. thaliana* WRKYs, and we named it a *Solanum* specific group. In Cluster2, the *Solanum* WRKYs were clustered together with the members of *A. thaliana* WRKY groups I, II, and III, and this group was named as a mixed group.

### Expansion or loss of WRKY gene family in tomato and its wild relatives

From a comparison between *V. vinifera* and six *Solanum* genomes, we got 50, 46, 39, 45, 48, and 45 WRKYs representing 56.82%, 59.74%, 48.75%, 52.94%, 56.47%, and 54.88% of corresponding total WRKYs in *S. lycopersicoides*, *S. pennellii*, *S. pimpinellifolium*, *S. lycopersicum* var. cerasiforme, *S. lycopersicum* cv. Heinz1706, and *S. lycopersicum* cv. M82 genomes, respectively. From the analysis of TD events for WRKYs in three tomatoes and their three wild relatives, we found that 10.2%, 7.8%, 10%, 7%, 11.8%, and 9.8% of total WRKYs in *S. lycopersicoides*, *S. pennellii*, *S. pimpinellifolium*, *S. lycopersicum* var. cerasiforme, *S. lycopersicum* cv. Heinz1706, and *S. lycopersicum* cv. M82 genomes respectively were generated by the TD event. These results demonstrated that the WGT event played an important role in the expansion of WRKY gene family in *Solanum* lineage. Phylogeny analysis of nine species revealed that Cluster1 contained *Solanum*-specific WRKY group, which meant that these WRKYs were generated after the WGT event of the Eudicot ancestor, leading to the expansion of WRKY gene family in six *Solanum* genomes. In the syntenic analysis between *V. vinifera* and six *Solanum* genomes, 44 WRKYs of 64 *V. vinifera* WRKYs were detected as orthologous WRKYs in *Solanum* lineage, which meant that the orthologous WRKYs of 20 *V. vinifera* WRKYs totally lost in six *Solanum* genomes after the WGT event of Eudicot ancestor. So, the WRKY gene family in *Solanum* genomes experienced complex retention or loss and formed the current WRKY gene family in *Solanum* lineage.

## Conclusion

Based on the highly conserved domain of WRKY gene family, we identified 33, 64, 48, 88, 77, 80, 85, 85, and 82 WRKY transcription factors in *A. trichopoda*, *V. vinifera*, *A. thaliana*, *S. lycopersicoides*, *S. pennellii*, *S. pimpinellifolium*, *S. lycopersicum* var. cerasiforme, *S. lycopersicum* cv. Heinz1706, and *S. lycopersicum* cv. M82, respectively. Through the analysis of WRKY distribution on *Solanum* genomes, there is no WRKY located on Chr11 pseudo-molecular chromosome in six *Solanum* genomes. Phylogenetic analysis indicated that all the WRKYs among nine genomes were divided into two different clusters, Cluster1 and Cluster2. These two clusters contained 194 and 448 WRKYs representing 30.22% and 69.78% of total WRKYs in nine genomes. From the analysis of WGT and TD events, we found that the WGT event brought a stronger influence on the expansion of the WRKY gene family in *Solanum* lineage. Expression analyses revealed that the expressed WRKYs in *S. lycopersicum* cv. Heinz1706 grouped into two clusters, and the kinds of paralogous WRKYs generated by WGT and TD events showed different expression patterns. This project is the first to illustrate the evolutionary history and expression characteristics of WRKYs in *Solanum* lineage, which will provide a novel view to study the expansion or loss of the WRKY gene family, and expression divergence of duplicated WRKYs in community.

## Data Availability

The datasets presented in this study can be found in online repositories. The names of the repository/repositories and accession number(s) can be found in the article/Supplementary Material.

## Abbreviations

HMM, Hidden Markov model; ML, Maximum likelihood; Myr, Million years; TD, tandem duplication; WGT, whole-genome triplication; WRKYs, WRKY transcription factors.
