# Supplementary figures and images for: Genome-wide analysis of the WRKY gene family unveil evolutionary history and expression characteristics in tomato and its wild relatives
Source: Front Genet. 2022 Sep 15;13:962975. doi: 10.3389/fgene.2022.962975 (PMC9520452; doi:10.3389/fgene.2022.962975)

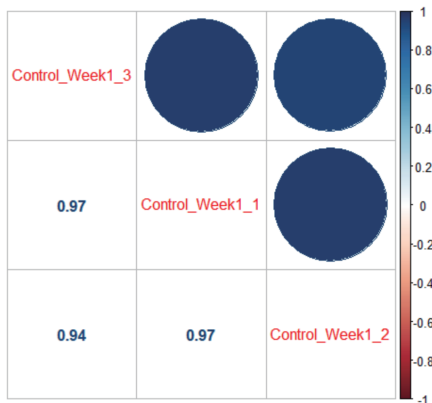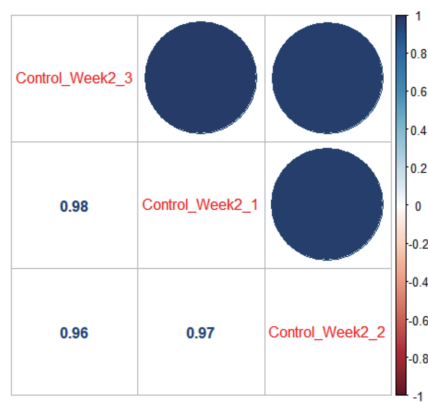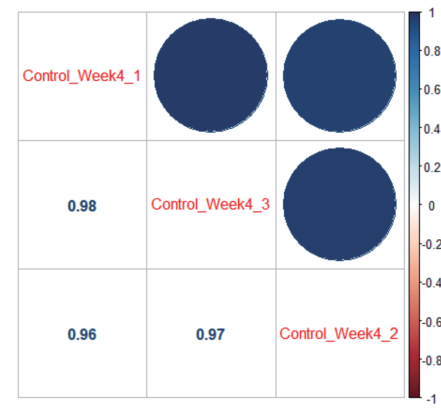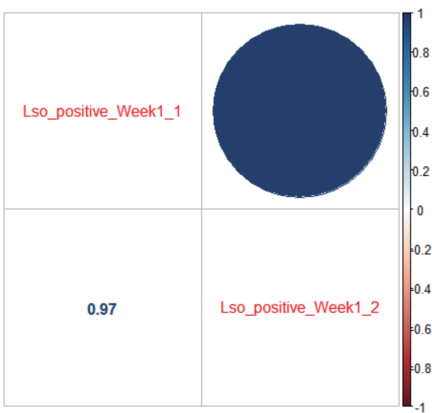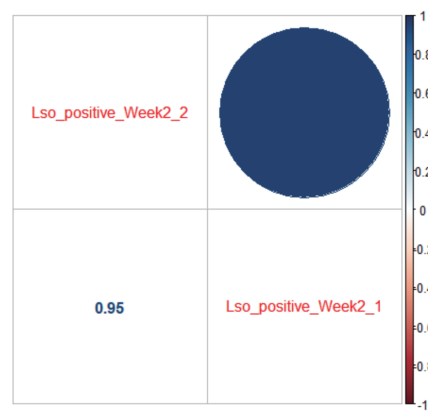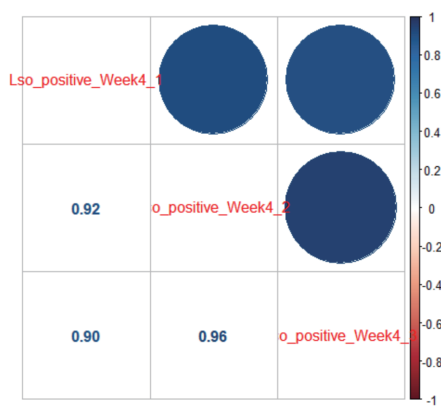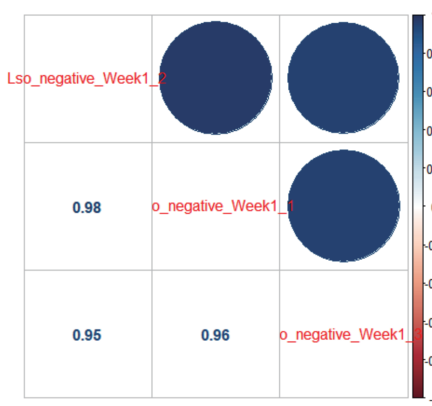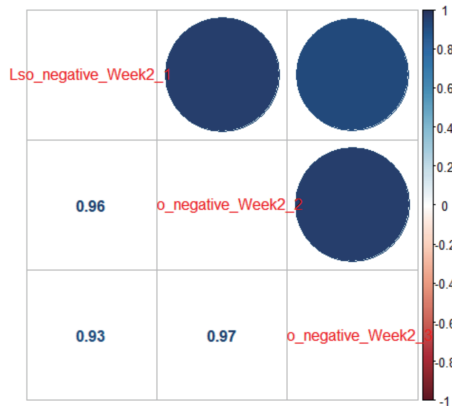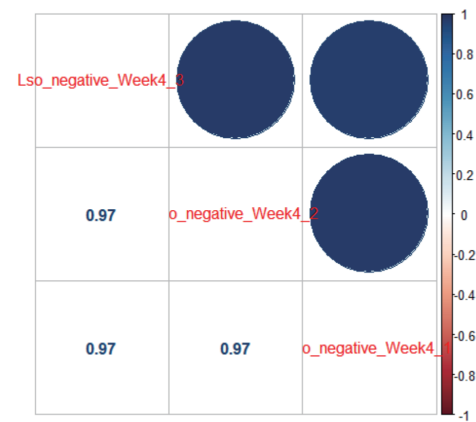

Supplement: Supplementary file 3 [file DataSheet1.PDF]
